# Supplementary material for: Cold storage-mediated rearing of Trichogramma evanescens Westwood on eggs of Plodia interpunctella (Hübner) and Galleria mellonella L
Source: PLoS One. 2021 Jun 14;16(6):e0253287. doi: 10.1371/journal.pone.0253287 (PMC8202947; doi:10.1371/journal.pone.0253287)
Supplement: Attachment — Submitted filename: Authors Response to Reviwers R2.docx [file pone.0253287.s002.docx]

**Response to Reviewers**

Reviewer #1: The quality of updated manuscript has been greatly improved based on the revision suggestions, but there are still serious problems on statistical analyses.

Response: Thanks for the appreciation

1. lines 184-186: The authors did not give the description in detail. For different trials, separately describe the statistical methods using ANOVA with multi-factors, what factors have been given for each trial.

Response: Revised the statistical methods as suggested. Pls see lines 182-190

2. Table 1: I'm still confused on some difference letters given in table, for example, for the results of adult emergence on 1 day age egg, the data of 87.50 and 91.67 with 'a' and 'ab', respectively. It should give the data: 91.67a, 87.5 ab. Carefully check all the statistical analysis results for Table 1, 2, and 3, then give the right letters for various treatments.

Response: The results for comparison letters presented in Tables 1, 2, 3 are checked and corrected accordingly

3. Fig. 1: I'm confused on all difference letters given on the bars. It is not clear to note "within each egg age, bars followed by the same letters are not significantly different; HSD test at 0.05". Checking Fig. 1, I'm confused on the indication. How you do the comparisions for two species at the same egg age, both togher or seperately? Similarly, the letters on the bars are also given in disorder, check all the statistical results carefully, then give the correct letters for various treatment.

Response: The results particularly for the comparison letters presented in Fig 1 are checked and corrected accordingly

4. Fig. 2: same to Fig. 1. particularly note: for 1 day old egg, at 15 d, why give the letter 'ad' for P. interpunctella?

Response: The results particularly comparison letters presented in Fig 2 are checked and corrected accordingly

Reviewer #2: Dear author

Thank you for reply all comments. You presented a very good argumentation; it was good to read it.

Response: Thanks for the appreciation
